# Multi-Toxic Endpoints of the Foodborne Mycotoxins in Nematode *Caenorhabditis elegans*

**DOI:** 10.3390/toxins7124876

**Published:** 2015-12-02

**Authors:** Zhendong Yang, Kathy S. Xue, Xiulan Sun, Lili Tang, Jia-Sheng Wang

**Affiliations:** 1 Synergetic Innovation Center of Food Safety and Nutrition, School of Food Science, Jiangnan University, Wuxi, Jiangsu 214122, China; zdyang777@163.com (Z.Y.); sxlzzz@jiangnan.edu.cn (X.S.); 2 Department of Environmental Health Science, University of Georgia, Athens, GA 30602, USA; ksxue@uga.edu (K.X.); jswang@uga.edu (J.S.W.)

**Keywords:** aflatoxin B_1_, *Caenorhabditis elegans*, deoxynivalenol, fumonisin B_1_, mycotoxins, T-2 toxin, zearalenone

## Abstract

Aflatoxins B_1_ (AFB_1_), deoxynivalenol (DON), fumonisin B_1_ (FB_1_), T-2 toxin (T-2), and zearalenone (ZEA) are the major foodborne mycotoxins of public health concerns. In the present study, the multiple toxic endpoints of these naturally-occurring mycotoxins were evaluated in *Caenorhabditis elegans* model for their lethality, toxic effects on growth and reproduction, as well as influence on lifespan. We found that the lethality endpoint was more sensitive for T-2 toxicity with the EC_50_ at 1.38 mg/L, the growth endpoint was relatively sensitive for AFB_1_ toxic effects, and the reproduction endpoint was more sensitive for toxicities of AFB_1_, FB_1_, and ZEA. Moreover, the lifespan endpoint was sensitive to toxic effects of all five tested mycotoxins. Data obtained from this study may serve as an important contribution to knowledge on assessment of mycotoxin toxic effects, especially for assessing developmental and reproductive toxic effects, using the *C. elegans* model.

## 1. Introduction

Mycotoxins are toxic secondary metabolites produced by fungi growing on agricultural commodities in the field or during storage [1,2]. These naturally-occurring mycotoxins display diverse chemical structures accounting for their differing biological properties and effects [3]. Depending upon their precise biochemical nature, they induced multiple toxic effects including carcinogenic, teratogenic, mutagenic, estrogenic, neurotoxic, and immunotoxic effects [4,5,6]. Spurred by the discovery of aflatoxins in the 1960s, more than 100 toxigenic fungi and in excess of 300 mycotoxins were identified worldwide [7]. The aflatoxins, fumonisins, ochratoxins, zearalenone (ZEA), and trichothecenes, such as deoxynivalenol (DON) and T-2 toxin (T-2) are the major foodborne mycotoxins of public health concerns [6,8].

Aflatoxins represent a group of closely related difuranocoumarin compounds mainly produced by *Aspergillus flavus*, *A. parasiticus*, and four naturally-occurring aflatoxins (B_1_, B_2_, G_1_, and G_2_) were identified. Aflatoxins have been found in a variety of agricultural commodities, but the most pronounced contamination has been encountered in maize, peanuts, cotton seed, and tree nuts with the levels ranged from 0.11 to 4030 µg/kg [9]. Aflatoxin B_1_ (AFB_1_) is the most prevalent and toxic, and is also known as being one of the most potent genotoxic agents and hepatocarcinogens [10,11]. Developmental and reproductive toxic effects and immunotoxic effects of AFB_1_ have recently been recognized in the research field.

Fumonisins are primarily produced by the fungi *Fusarium* *verticillioides* [12]. The most common fumonisin found in maize is fumonisin B_1_ (FB_1_), while fumonisins B_2_ and B_3_ (FB_2_ and FB_3_) are common co-contaminants. FB_1_ was mainly found in corn and corn products with the levels ranging from 0.01 to 330 mg/kg [13]. FB_1_ exposure was suggested to link to a broad spectrum of animal and human diseases, such as hepatocellular carcinoma and esophageal cancer in South Africa, China, and the Islamic Republic of Iran [14,15,16,17,18]. In addition to the carcinogenic property, FB_1_ exposure played a role in the occurrence of a cluster of neural tube defects along the Texas-Mexico border [19].

ZEA is mainly produced by *Fusarium* *graminearum* and is primarily contaminated maize but occurs in modest concentrations in wheat, barley, and sorghum, and concentrations in food ranged from 0.01 to 2909 mg/kg [20]. ZEA induces genotoxicity, immunotoxicity, developmental and reproductive toxicities, and tumorigenicity in various animal models [21,22]. Due to its structural similarity to estrogen, ZEA may bind to human estrogen receptors and elicit permanent reproductive tract alterations [23]. Studies also suggested that ZEA exposure was associated with a high incidence of primary liver cancer in animals and human and may contribute to the increasing occurrence of breast cancer.

DON and T-2 are trichothecene mycotoxins produced by many fungi genera and plants [24] and over 80 diverse compounds in structure were found [25]. DON is probably the most widely distributed *Fusarium* mycotoxin in cereals and its contamination was reported in various crops and processed grains with the range from 0.01 to 500 mg/kg [20]. T-2 has been reported in cereals in many parts of the world with the range from 0.01 to 40 mg/kg, and it is formed in large quantities under the unusual circumstance of prolonged wet weather at harvest [26]. These two mycotoxins had significant pathophysiological effects in humans and animals because of their interference with protein synthesis [27]. DON and T-2 induced phosphokinase-mediated stress pathways, aberrantly activated proinflammatory gene expression, disrupted gastrointestinal function and growth hormone action, and caused cell death [28]. Acute exposures to high doses of T-2 or DON in experimental animals induced anorexia, diarrhea, and vomiting; moreover, at extremely high doses, lethal toxic effects were observed, including gastrointestinal hemorrhage, leukocytosis, circulatory shock, reduced cardiac output, and ultimately, death. Chronic exposure of animals to moderate doses impaired food intake, reduced weight gain, disrupted immune function, and caused developmental toxic effects [28].

Due to the widespread nature of toxigenic fungi in the environment, mycotoxins were considered as unavoidable contaminants in foods and feeds; therefore, one of the most effective measures to protect public health is to set up regulatory levels of these toxins. Well-defined toxic endpoints and mechanistic studies are the basis for establishing regulatory level and risk assessment. Although rodent-based assays have been the traditional models for toxicological studies, it is well-recognized that rodent assays are time consuming and expensive. Therefore, other *in vivo* or *in vitro* assays were introduced into toxicological field to take their advantages for rapid, inexpensive, and without concern of animal welfare issues. Although *in vitro* cell-based assays are commonly used, whole organism models are biologically relevant and allow observing both genomic alterations and phenotypic modifications. The nematode *Caenorhabditis elegans* (*C. elegans*), a popular model organism for genetic and developmental biology research [29], is now being recognized as an attractive invertebrate model for toxicological studies [30,31,32,33,34,35].

*C. elegans* is a free-living nematode that has some attractive properties, such as short life cycle (3–4 days), ease of culturing, and low cost. Its well-defined genome, completed cell lineage map, knockout (KO) mutant libraries, and well established methodologies including mutagenesis, transgenesis, and RNA interference (RNAi) can provide a variety of options to manipulate molecular mechanisms. Furthermore, there is a high degree of conservation between *C. elegans* and mammalian species in processes controlling development, neurobiology, and stress responses [36]. For these unique features *C. elegans* met with the “Three R” requirements (replacement, reduction, and refinement) and has been extensively applied to toxicological fields in the recent years [37,38]. Nevertheless, there are fewer studies in the literature for studying toxic effects of foodborne mycotoxins in *C. elegans*. Evidence that could link *C. elegans* with mycotoxin toxicities in humans included the presence of CYP450 orthologue, which can metabolize AFB_1_ similar to in humans, as well as various orthologues of glutathione transferase, one of the most well-known phase II metabolic and detoxification mechanisms of AFB_1_. Furthermore, there is a high degree of conservation between *C. elegans* and mammalian species in processes controlling development, neurobiology, and stress responses, which allow us to explore molecular mechanisms of reproductive, developmental, and transgenerational effects of mycotoxins.

**Figure 1:**
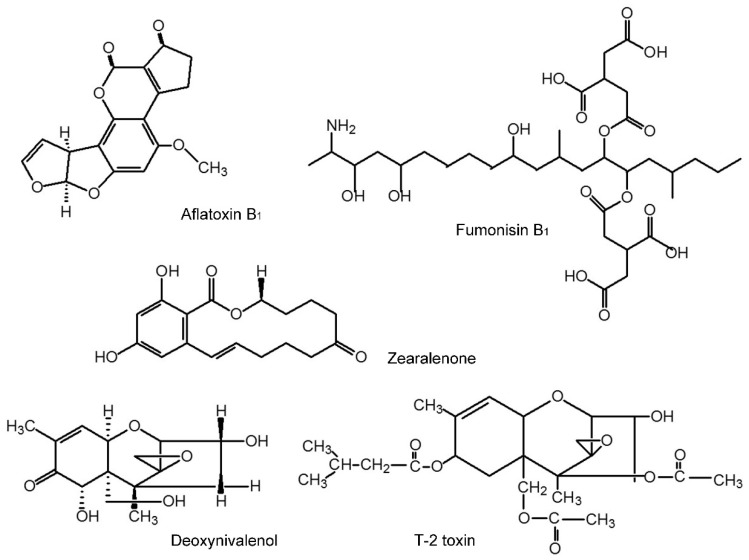
The structural formula of five mycotoxins.

In this study, we investigated multiple toxic endpoints of common foodborne mycotoxins, AFB_1_, DON, FB_1_, T-2, and ZEA with structures shown in Figure 1, in *C. elegans* model, including lethality, toxic effects on growth and reproduction as well as influence on lifespan. Data presented clearly demonstrated that *C. elegans* model can predict toxic effects of mycotoxins, and can use for mechanistic studies of mycotoxins-induced adverse health effects.

## 2. Results

### 2.1. Lethality

The wild-type N2 strain was treated with various concentrations of tested mycotoxins for 24 h and their LC_50_ values were calculated and shown in Table 1. Lethality in control worms was less than 10% in all cases. The potency for lethality, as represented by LC_50_ values, in the wild-type N2 worms was T-2 > AFB_1_ > ZEA > FB_1_ > DON. Toxicity ranking based on LC_50_ is T-2 (1 mg) > AFB_1_ (20 mg) > ZEA (76 mg) > FB_1_ (235 mg) > DON (657 mg).

**Table 1:** Lethality for wild-type N2 *C. elegans* treated with mycotoxins.

Mycotoxins	AFB1	DON	FB1	T-2	ZEA
LC_50_ mg/L	20.47	656.67	235.62	1.38	75.79
95%CI	12.67–45.21	435.96–1145.66	79.07–640.142	1.01–1.76	5.83–985.03

### 2.2. Toxic Effects on Growth

**Figure 2:**
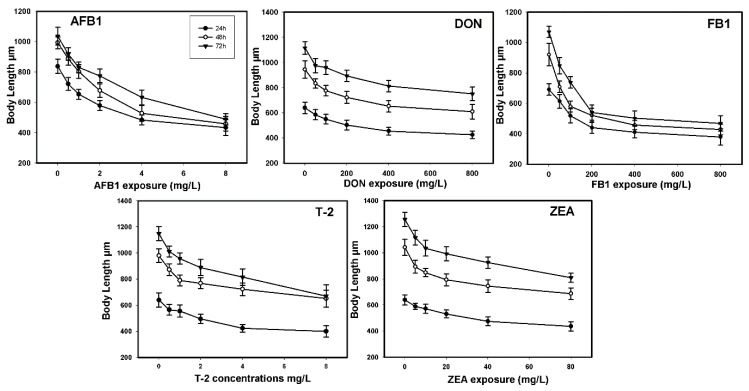
Effects on Body length of *C. elegans* exposed to mycotoxins at 24 h, 48 h, and 72 h.

**Figure 3:**
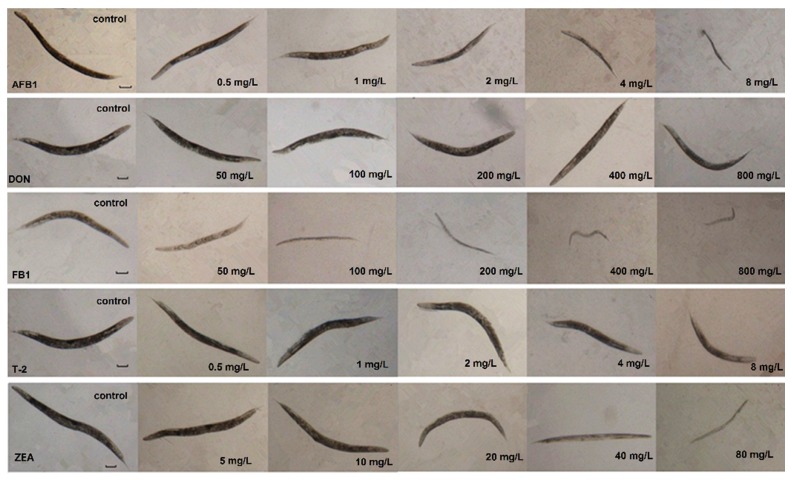
Morphological changes of adult *C. elegans* following 72 h exposure to mycotoxins (scale in 100 µm for all panels).

As shown in Figure 2, five mycotoxins affected the growth of worm as indicated by body length, in dose-effect (*p* < 0.01) and time-effect manner (*p* < 0.05). Following 72 h exposure, AFB_1_ and T-2 at the concentration of 8 mg/L caused the greater growth-inhibitory effects, reaching 52.8% and 41.61% size reduction than untreated controls (*p* < 0.001). The median effective concentrations (EC_50_) of AFB_1_ and the T-2 was 7.31 mg/L (95%CI: 5.19–12.9 mg/L) and 16.91 mg/L (95%CI: 9.31–59.81 mg/L), which was 300 times lower than that of ZEA and FB_1_. Similar to what found in lethality testing, DON did not result in significant growth inhibitions at the concentrations between 50 mg/L and 800 mg/L (Table 2). The morphological changes caused by exposure to these five mycotoxins at 72 h were shown in Figure 3.

**Table 2:** Toxic effects on growth and reproduction in N2 *C. elegans* following 72 h exposure to mycotoxins.

Mycotoxins	Growth			Reproduction		
	EC_50_ mg/L	Interval of Confidence (95%)	Ratio LC_50_/EC_50_	EC_50_ mg/L	Interval of Confidence (95%)	Ratio LC_50_/EC_50_
AFB_1_	7.31	5.19–12.50	2.80	1.69	1.38–2.04	12.11
DON	533.07	412.63–686.02	0.49	487.28	311.15–515.90	0.55
FB_1_	361.59	261.36–569.77	0.65	25.63	19.63–31.77	9.19
T-2	16.96	9.13–59.81	0.08	1.82	1.33–2.44	0.76
ZEA	314.19	129.74–860.21	0.24	26.05	19.23–37.17	2.90

### 2.3. Toxic Effects of Reproduction

**Figure 4:**
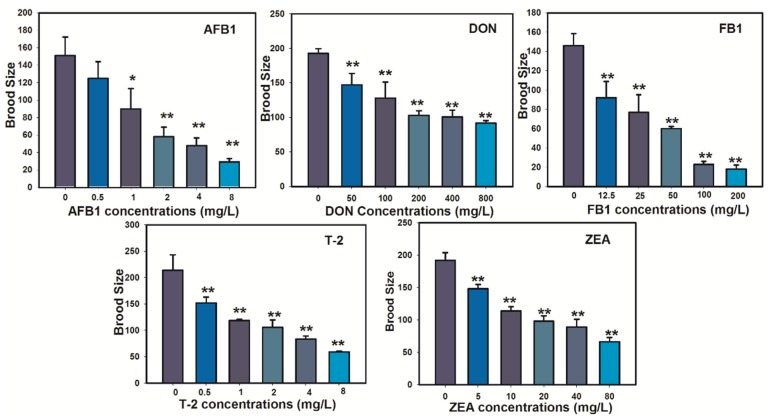
Toxic effects on brood size of N2 *C. elegans* following 72 h exposure to mycotoxins.

Toxic effects on the N2 nematode reproduction, represented by number of offspring (brood size), as a function of mycotoxins concentration were plotted and shown in Figure 4. The average number of offspring for the untreated controls was 133 ± 22, comparable to previous studies [39]. The brood size was significantly reduced in at all tested concentrations for AFB_1_ (*p* < 0.001), DON (*p* < 0.05), FB_1_ (*p* < 0.001), T-2 (*p* < 0.001), and ZEA (*p* < 0.001), respectively, as compared to that in the untreated controls. Reproductive effects were commonly detectable at much lower concentrations of FB_1_, which suggested that *C. elegans* is a much sensitive model for testing reproductive toxic effects of FB_1_ as compared to other lethality and growth endpoints. The EC_50_ value with 95% CI was estimated from the concentration-effect curve of each treated mycotoxin and listed in Table 2. The most sensitive mycotoxin is AFB_1_ with EC_50_ of 1.69 mg /L (95% CI, 1.38–2.04 mg/L).

### 2.4. Influence on Lifespan

**Figure 5:**
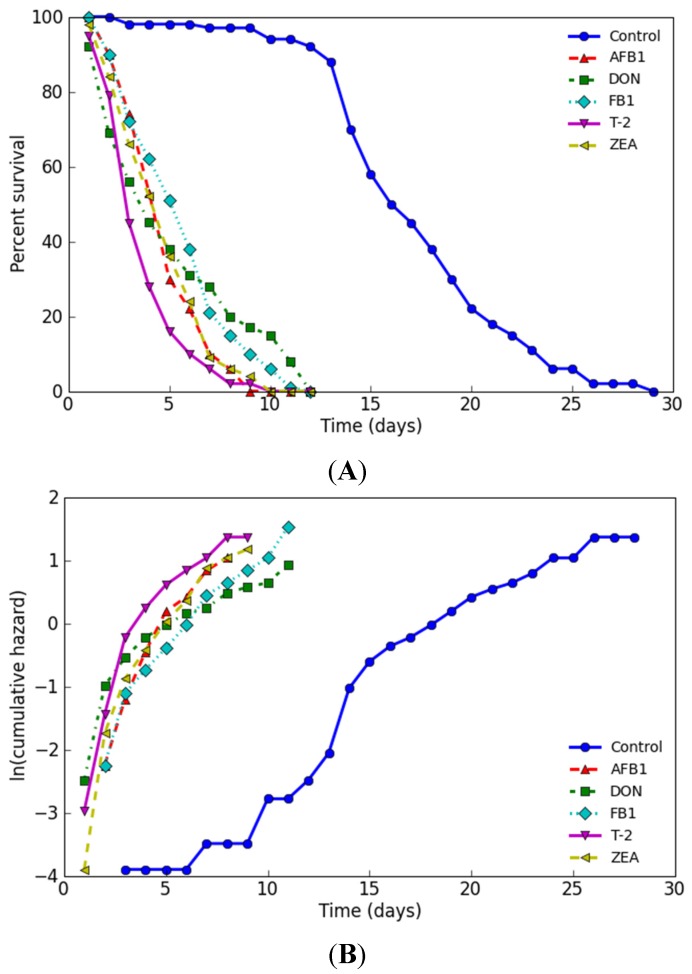
Survival curves (**A**) and log cumulative hazard plots (**B**) in N2 *C. elegans* treated with tested mycotoxins.

The lifespan of N2 nematode treated with 10% LC_50_ of five tested mycotoxins was independently recorded in order to test and compare the sensitivity of the assay. Mycotoxin treatment decreased lifespan and increased mortality rate, as illustrated by survival curve (Figure 5A), log cumulative hazard plots (Figure 5B), and data in Table 3. The survival curves of five mycotoxins were shifted to the left compared to untreated controls. The shape of the cumulative hazard plots, which reflected the rate of aging [40], and the y-intercept of the log cumulative hazard plots of five mycotoxins were significantly larger than that of untreated control (*p* < 0.0001) as assessed by OASIS. The mean lifespan exposed to AFB_1_, DON, FB_1_, T-2, and ZEA significantly decreased from 17.26 ± 0.47 days to 4.85 ± 0.19, 5.19 ± 0.35, 5.66 ± 0.26, 3.83 ± 0.29, and 4.79 ± 0.22 days, respectively (*p* < 0.0001). As compared to the untreated control, the median lifespan time was significantly decreased by 70.59%, 57.65%, 62.94%, 80.59%, and 68.82%, respectively.

**Table 3:** Influence on lifespan in N2 *C. elegans* treated with five tested mycotoxins.

Name	Dose (mg/L)	No. of Subjects	Estimated Mean			Mortality (Days)				
			Days	Std.	95% C.I.	25%	50%	75%	90%	100%
**Control**	0	100	17.26	0.47	16.34–18.18	14	16	20	24	29
**AFB_1_**	2.05	100	4.85	0.19	4.48–5.22	3	5	6	7	9
**DON**	65.67	100	5.19	0.35	4.50–5.88	2	4	8	11	12
**FB_1_**	23.56	100	5.66	0.26	5.15–6.17	3	6	7	9	12
**T-2**	0.14	100	3.83	0.19	3.46–4.20	3	4	5	6	10
**ZEA**	7.58	100	4.79	0.22	4.36–5.22	3	5	6	7	10

## 3. Discussion

*C. elegans* has become a popular toxicity test organism, as well reviewed in details [32,34,41]. Much of the early work explored metal toxicity and used lethality as the major endpoint [42]. A wider variety of toxicants have been tested with *C. elegans* in recent years and more sophisticated sub-lethal endpoints have been developed, including parameters for growth and reproduction [30]. These types of endpoints were directly applied for evaluating environmental toxicants and used as an alternative method for mammalian testing [43]. There were fewer studies in the literature devoted to assess foodborne mycotoxins toxicity using *C. elegans*. Leung *et al.* [44] found that AFB_1_ induced toxic effects on growth and reproduction in *C. elegans* at the concentrations of 3, 30, and 100 µM, respectively. The progeny production and development rates of the nematode were significantly reduced when treated with DON at concentrations of 500 and 1000 mg/L [45]. Our present study showed that LC_50_ values of tested mycotoxins were at very high concentrations with the exception of T-2 (1.38 mg/L) and AFB_1_ (20.47 mg/L). These findings were consistent with results using other model systems [46,47]. Compared to LD_50_ or LC_50_ values obtained from other model systems such as rats, fish, and human cells in the literature [1,6,48,49] similar acute toxic response was found between rats and *C. elegans* for T-2. Similar or less acute toxic response for AFB_1_ was found in *C. elegans* (20 mg/L) as compared to values in rats (2.7–17.9 mg/L). *C. elegans* model is more sensitive for ZEA and less sensitive for DON as compared to LD_50_ in rodent model. It is hard to make conclusion for FB_1_ because no LD_50_ is available in rodents.

Following 72 h exposure, five mycotoxins had significant inhibitory effects on growth and reproduction endpoints of the nematode. Similar to what found for LC_50_, AFB_1_ and T-2 had greater inhibitive effects than other tested mycotoxins on growth and reproduction. DON had minimal effects as compared to other four mycotoxins. The LC_50_ and EC_50_ (growth and reproduction) values (Table 2) were compared to evaluate the sensitivity of these toxic endpoints. As anticipated, large differences between lethality values and effective concentrations of growth or reproduction were found. In the case of AFB_1_, the LC_50_/EC_50_ ratio was 2.8 for growth and 12.11 for reproduction, which indicated that the EC_50_ of growth and reproduction values are more sensitive than the LC_50_ value. Thus, endpoints of growth and reproduction would be much more sensitive indicators of AFB_1_ toxicity than endpoints of lethality. Same cases for reproductive toxicity in FB_1_ and ZEA were found. On contrary, the lethality endpoint was more sensitive for T-2 than other mycotoxins and none of these three endpoints was sensitive for DON. Findings in our study were consistent with the reports in other species [50].

Traditionally, the lethality assay was a standard toxicity assay of *C. elegans* model, and the advantage of the assay was the relative ease in scoring worms’ mortality and analysis. However, some mycotoxins so far tested, e.g., DON, were not very sensitive to the lethality endpoint, because mycotoxins are secondary metabolites of fungi and their toxic effects cumulated over a period of time [6] in addition to their different target organs and mode of actions. The less sensitive to DON was also observed in the earthworm [50].

Lifespan, rather than physiological indicators, is resulted from complex interactions between genetic, environmental, and stochastic factors and can provide critical insights into the entire life cycle affected by xenobiotics, including mycotoxins. As shown in our data, all five tested mycotoxins could result in shortening lifespan and increase mortality rate in *C. elegans*. The median lifespan time was significantly decreased following treatment with mycotoxins. These results suggested that influence on lifespan may be a specific endpoint for testing toxic effects of environmental toxicants like mycotoxins. In comparison with most of the other species currently used, lifespan assessment with *C. elegans* has been simplified and is easy to detect using a microscope, and to analyze with the established software [35]. In summary, we evaluated multiple endpoints for testing mycotoxin toxicities. Lethality endpoint was more sensitive for T-2 toxicity. The toxicity ranking for LC_50_ is T-2 (1 mg/L) > AFB1 (20 mg/L) > ZEA (76 mg/L) > FB_1_ (235 mg/L) > DON (657 mg/L). Reproduction endpoint was more sensitive for toxicities of AFB_1_, FB_1_, and ZEA. The ranking for reproduction: AFB_1_ (2 mg/L) = T-2 (2 mg/L) > ZEA (26 mg/L) = FB_1_ (26 mg/L) > DON (487 mg/L). The growth endpoint was also sensitive for AFB_1_ toxicity. The ranking for growth: AFB_1_ (7 mg/L) > T-2 (17 mg/L) > ZEA (314 mg/L) ≥ FB_1_ (362 mg/L) > DON (533 mg/L). Moreover, the lifespan endpoint was sensitive to test toxic effects of all five mycotoxins. Data obtained from this study may serve as an important contribution to knowledge on evaluation of toxic effects of mycotoxins using *C. elegans* model, especially for assessing developmental and reproductive toxic effects of mycotoxins exposure in humans and animals.

## 4. Experimental Section

### 4.1. Materials

Mycotoxins selected for this study, including aflatoxin B_1_, deoxynivalenol, fumonisin B_1_, T-2 toxin, and zearalenone, were purchased from Sigma-Aldrich Chemical Co. (St. Louis, MO, USA). Purity of each toxin (95%–99%) was tested with the appropriate analytical tools (HPLC, LC/MS, and GC/MS). Stock solutions were made with dimethylsulfoxide (DMSO) and kept under argon. Worms used in the present study, wild-type Bristol (N2), and *E. coli* strain OP50 were purchased from the *Caenorhabditis* Genetics Center (Minneapolis, MN, USA). All the worms used in this research were hermaphrodites. Worms growth medium (NGM) was made as previously described by Brenner [29]. All other chemicals and reagents were purchased commercially at the highest degree of purity available.

### 4.2. Mycotoxins Exposures

Five mycotoxins in stock solutions were diluted to different concentrations of test solutions. Three- to four-day old worms were dispensed into each well of a 12-well plate. Each well contained a mixture of 990 μL complete K-medium, 10 μL test solution, and OP50. The 1% DMSO was found not affecting nematode growth or reproduction (data not shown). The exposure concentrations were selected based on preliminary lethality assays or solubility testing in complete K-medium with 1% DMSO, e.g., AFB_1_ had solubility limits of ~50 mg/L K-medium.

### 4.3. Lethalality Assay

All worms were cultured at 20 °C in Petri dishes. Lethality tests were performed on the three-day old wild-type worm for 24 h exposure to different concentrations of mycotoxins using methods described by Donkin and Williams [51]. Briefly, each test consisted of five concentrations plus a control, in which 10 ± 1 worms (30 worms for each concentration) were transferred to 12-well tissue culture plates containing 1 mL of the test solution in each of five wells. Mycotoxins solutions were prepared in K-medium (0.051 M NaCl and 0.032 M KCl) [52], because worms suffer osmotic stress in deionized water. At the end of the exposure period, worms were counted and scored as live or dead under a microscope; they were judged to be dead if they did not respond to touch using a small, metal wire. All experiments were repeated for three times and the LC_50_ values were derived through a Probits analysis.

### 4.4. Measurement of Growth Endpoint

Growth was assessed by measuring change in body length over a 72 h exposure period. The synchronized L-2 worms were used to develop at 20 °C either in control or five mycotoxins at different concentrations in K-medium with food. After exposure 72 h, 20 worms were mounted into a glass pad containing 10% formalin solution. Body length analysis (head to tail) was performed using an Olympus SZX9 microscope (Olympus America Inc. Center Valley, PA, USA) and Infinify analyze software (V5.0.2, Lumenera Corporation, Ottawa, ON, Canada, 2009). Three independent experiments were performed and, for each experiment, at least 20 control and treated worms were analyzed.

### 4.5. Measurement of Reproductive Endpoint

Reproduction was tested using the 72 h assays described by Dhawan *et al.* [42]. The test solutions consisted of different concentrations of AFB_1_ (0–8 mg/L), or DON (0–800 mg/L), FB_1_ (0–800 mg/L), T-2 (0–8 mg/L), ZEA (0–80 mg/L), respectively. One adult worm from an age-synchronized culture was placed in each 1 mL of test solution. Three wells were used for each concentration and exposed under the same conditions as described for the growth test. Three days later, the number of offspring at all stages beyond the eggs was determined [39]. For each test concentration and control, the average number of progeny from three wells was obtained for each test replicate, and the testing was repeated three times.

### 4.6. Life-Span Experiment

Lifespan analysis was conducted at 20 °C as described previously [53]. Synchronized young adult worms were placed on NGM agar plates and treated with 10% LC_50_ of five tested mycotoxins and 0.1 mg/mL of 5-fluorodeoxyuridine (5-FUDR, Sigma, St. Louis, MO, USA) which was used to block progeny development [54]. Control experiments indicated that 5-FUDR did not affect worms’ lifespan. Worms were transferred to fresh treatment plates every other day, and scored every day by gentle prodding with a platinum wire to test for live or dead worms. Those had ceased pharyngeal pumping and failed to move, even after repeated prodding, were scored as dead and removed from the plates. Worms that had crawled off onto the sides of the plate and died away from the agar were censored. A minimum of 100 worms was counted and scored per condition per experiment. Lifespan was defined as the time elapsed from when worms were put on treatment plates to when they were scored as dead. Three independent life-span studies were performed.

### 4.7. Statistical Analysis

The concentration-response relationships for lethality, growth, reproduction, and lifespan were generated from three independent replicate tests. The median lethal concentration (LC_50_) and median effective concentration (EC_50_, concentration producing a 50% reduction in body size or offsprings compared to control) with 95% confidence intervals (CI), were calculated using logistic regression. Response variables that were not normally distributed were transformed by logarithmic to improve normality. Generalized Linear Models (GLMS) was used to evaluate the significant difference among treatments and between all treatment levels and the control. The SAS 9.4 (SAS Institute, Cary, NC, USA) was used for data analysis and a *p*-value of 0.05 or less was considered to be statistically significant.

To determine the effects of experimental treatments on survival, a comprehensive comparison of survival datasets between an experimental group and a control group was analyzed using OASIS (online application of survival analysis, http://sbi.postech.ac.kr/oasis) [49]. The average survival time was obtained by using log-rank test, whereas those of a specific time point can be obtained by using Fisher’s exact test [13]. If two data sets at 90% mortality show no statistically significant, the weighted log-rank test was used instead of Log-rank test, which developed by Fleming and Harrington [48] and was sensitive to early differences.

## 5. Conclusions

We evaluated multiple toxic endpoints for five common foodborne mycotoxins. Lethality endpoint seemed more sensitive for T-2 toxicity. Reproduction endpoint was more sensitive for toxicities of AFB_1_, FB_1_, and ZEA. Growth endpoint was also sensitive for AFB_1_ toxicity. Moreover, lifespan endpoint was sensitive to test toxic effects of all five mycotoxins. Data obtained from this study suggests *C. elegans* model can serve as a good model organism for evaluation of toxic effects of mycotoxins, especially for assessing developmental and reproductive toxic effects.
